# Factors Associated with Prenatal Health Behaviors among Low-Income, Ethnic Minority Women

**DOI:** 10.3390/ijerph20031695

**Published:** 2023-01-17

**Authors:** Guido G. Urizar, Joshua Murillo, Karissa Miller

**Affiliations:** Department of Psychology, California State University, 1250 Bellflower Blvd, Long Beach, CA 90840-0901, USA

**Keywords:** pregnancy, ethnic minority, health behaviors, coping, stress

## Abstract

Less than one-third of pregnant women in the U.S. meet prenatal nutrition, exercise, and stress management health behavior guidelines. Low rates of these prenatal health behaviors have been especially observed among low-income, ethnic minority women, placing them and their infants at a disproportionally higher risk for health complications. Yet, few studies have identified factors associated with these prenatal health behaviors in this population. This study examined whether certain demographic (e.g., ethnicity) and psychosocial characteristics (i.e., coping, stress, pregnancy-specific stress, and depression) were associated with prenatal nutrition (i.e., high-fat food and fruit and vegetable intake), exercise, and stress management health behaviors in 100 low-income, pregnant women (39% African American, 30% foreign-born Latinas, 15% U.S.-born Latinas, 10% non-Hispanic white, and 6% Asian American/Pacific Islander) in southern California using an embedded, mixed-methods, cross-sectional design. Results demonstrated that ethnic minority women who experienced more stress and used more maladaptive coping strategies (e.g., avoidance) were particularly at risk of consuming more high-fat foods and engaging in less exercise and stress management during pregnancy. Qualitative responses revealed women’s experiences with these prenatal health behaviors. These findings highlight the need for interventions and collaborative care models that target psychosocial factors in order to optimize prenatal health behaviors and health outcomes among ethnic minority women.

## 1. Introduction

Prenatal health behaviors play an important role in shaping the health of pregnant women and their developing fetuses. Specifically, unhealthy dietary patterns (high-fat food/low fruit and vegetable intake), sedentary behavior, and poor stress management during pregnancy have been found to increase a woman’s risk for gestational diabetes, hypertension, preterm labor, and low infant birthweight [1,2,3,4]. Given this evidence, the American College of Obstetricians and Gynecologists (ACOG) released nutrition and exercise guidelines recommending that pregnant women limit their fat intake to 20–35% of their total calories, consume two servings of fruits and two to three servings of vegetables daily, and engage in 30 min or more of moderate-intensity exercise on most days of the week [5,6]. Further, the ACOG recommended that women be screened for psychosocial stressors throughout pregnancy to be referred to stress management services when needed by their primary health and obstetric care providers [7]. Engagement in these health behavior guidelines has been associated with fewer prenatal (e.g., gestational diabetes and anxiety) and birth complications (e.g., preterm births and low birthweight) for both mothers and their infants [8,9,10]. Despite these guidelines, merely one-third of pregnant women in the U.S. (35–38%) meet the ACOG’s nutrition and exercise recommendations [11,12], with less than 25% actively engaging in stress management during pregnancy and only 14% of pregnant women with mental health symptoms being referred for stress management by their health care providers [13,14]. These rates are even lower among low-income and ethnic minority women despite their disproportionately higher number of pre- and postnatal health complications, such as hypertension, preterm labor, low birthweight, low APGAR scores, premature birth, and increased incidence of infant hospitalizations [15,16,17,18,19]. Given the heightened health concerns among low-income, ethnic minority pregnant women, studies are needed to identify factors associated with prenatal nutrition, exercise, and stress management behaviors in this population.

One theoretical model used to identify factors associated with health behaviors is the transactional model of stress and coping. According to this model, variations in the adoption of health behaviors are largely dependent on contextual factors, such as the presence of certain demographic characteristics (e.g., low socioeconomic status and ethnicity), one’s level of stress, and the strategies used to cope with stressors [20]. Studies have supported this multidimensional model demonstrating that low-income women and those experiencing elevated levels of stress, pregnancy-specific stress, and depression engage in fewer positive health behaviors during pregnancy [21,22,23]. Ethnic differences in prenatal health behaviors have shown mixed results, with some studies revealing African American women to have higher rates of fast-food consumption and lower rates of exercise during pregnancy than non-Hispanic white women [16,18,24]. Similarly, studies show that U.S.-born Latinas consume more fast food and fewer fruits and vegetables than foreign-born Latinas [17]. Coping responses, which involve the use of several cognitive and behavioral strategies to manage stressful situations, can also play a major role in pregnant women’s health behaviors. However, very few studies have examined the role of coping on prenatal health behaviors, with one recent study in Iran suggesting that lower use of adaptive coping strategies (e.g., planning) and greater use of maladaptive coping strategies (e.g., avoidance) are associated with poor nutrition and less exercise during pregnancy [25]. These results highlight the need to further examine the impact of these coping strategies on prenatal health behaviors in populations at risk for health complications during pregnancy.

### Present Study

The purpose of the current mixed-methods study was to describe the health behaviors of low-income, ethnic minority women during pregnancy, as well as to identify whether certain demographic and psychosocial characteristics (i.e., coping, stress, and depression) were associated with health behaviors in this population. Qualitative interviews were also administered to provide more in-depth information on the health behaviors these women engaged in during pregnancy.

## 2. Methods

### 2.1. Participants

Women were recruited from several prenatal clinics and community health organizations in southern California (between September 2008 and May 2010) as part of the Healthy Mothers, Healthy Babies Project, either through research staff who approached them in waiting rooms during their prenatal clinic visits, or through print-based advertising (brochures, flyers, and online ads) and referrals from their health care providers. These recruitment sites serve a predominantly low-income population (38% below poverty level; [26]). A quota-sampling strategy was used to provide an ethnically diverse sample of African American, Latina, Asian American/Pacific Islander, and non-Hispanic white women that was representative of the surrounding communities. Women signed consent forms to determine their study eligibility and to participate in the study. Eligibility criteria included being 18 years of age or older, between 10 and 24 weeks pregnant, fluent in either English or Spanish, and free of any major medical or psychiatric disorders (e.g., gestational diabetes and major depression).

### 2.2. Procedures

Eligible women (*n* = 100) participated in a one-hour health interview (using a mixed-methods, embedded, cross-sectional study design) conducted in English or Spanish (based on the participant’s preference) that assessed their demographic characteristics, use of different coping strategies, level of stress and depression, and health behaviors once during early pregnancy (participants were between 10 and 24 weeks pregnant at the time of the interview). A representative subset of 40 women from the larger sample (stratified by ethnicity) was randomly selected to participate in a separate qualitative interview to obtain more in-depth information on the health behaviors they engaged in during pregnancy. All study procedures were approved by the Institutional Review Board at California State University, Long Beach.

### 2.3. Measures

*Demographic Characteristics*. The demographic questionnaire assessed for maternal characteristics, such as age (in years), total years of education, annual household income, ethnicity (African American, foreign-born Latina, U.S.-born Latina, Asian American/Pacific Islander, or non-Hispanic white), marital status (single or married/living together), body mass index (kg/m^2^), gestational age (in weeks), parity, and planned pregnancy (yes/no).

*Coping Strategies*. Planning and avoidance coping were assessed using two subscales from the revised version of the Prenatal Coping Inventory (PCI; [27]), and receipt of social support was assessed using the Prenatal Social Support Questionnaire (PSSQ; [28]). The 8-item planning subscale measures the degree to which women plan for their baby’s arrival, and the 7-item avoidance coping subscale examines the degree to which women avoid or escape from the reality of the pregnancy. Items are rated on a 5-point scale of 0 (‘Never’) to 4 (‘Very often’). Planning (range = 0–32) and avoidance coping (range = 0–28) are calculated by summing the items corresponding to each subscale, with higher scores representing greater use of that coping strategy. The 12-item PSSQ asks women to indicate how often they perceive having received emotional, instrumental, and financial support from others during their pregnancy on a 4-point scale of 1 (‘I never feel this way’) to 4 (‘This is exactly how I feel’), with higher scores (range = 12–48) representing receipt of greater social support. Both the PCI [27] and the PSSQ [28] have been shown to have good reliability and validity. The Cronbach’s alpha for the current study was 0.84 (planning subscale) and 0.80 (avoidance coping subscale) for the PCI and 0.81 (social support) for the PSSQ.

*Stress*. Stress was assessed using the four-item version of the Perceived Stress Scale (PSS-4; [29]). The PSS-4 measures the extent to which situations in one’s life are perceived as stressful, unpredictable, and uncontrollable over the past month. Items are rated on a 5-point scale of 0 (‘Never’) to 4 (‘Very often’). A total score was calculated by reverse-coding two of the positively worded items and then summing all four items, with higher scores (range = 0–16) representing higher levels of stress. A cut-off score of 8 represents high stress [29]. The PSS-4 has been well-validated and has been shown to have good internal consistency when assessed within a one-month timeframe [27]. The Cronbach’s alpha for the current study was 0.69.

*Pregnancy-Specific Stress*. Pregnancy-specific stress was assessed using the 20-item version of the Pregnancy Experience Scale (PES-brief; [30]). The PES-brief is a measure of maternal exposures to daily, ongoing hassles (10 items) and uplifts (10 items) specific to pregnancy that are rated on a 4-point Likert scale of 0 (‘Not at all’) to 3 (‘A great deal’). Scores for both a frequency ratio (dividing number of hassles by number of uplifts endorsed) and an intensity ratio (dividing hassle intensity scores by uplift intensity scores) were derived, with higher frequency ratio scores (range = 0–10) indicating that women experienced a higher number of hassles relative to uplifts and higher intensity ratio scores (range = 0–3) indicating that women experienced more intense hassles relative to their uplifts. The PES-brief has been shown to have good reliability and validity [30]. The Cronbach’s alpha for the current study was 0.83 for the frequency ratio and 0.89 for the intensity ratio.

*Depression*. Depression was measured using the 10-item version of the Center for Epidemiologic Studies Depression Scale (CESD-10; [31]). The CESD-10 assesses for affective and somatic symptoms of depression during the previous week. Items are rated on a 4-point scale of 0 (‘Rarely’) to 3 (‘A majority of the time’). A total score was calculated by reverse-coding two of the positively worded items and then summing all 10 items, with higher scores (range = 0–30) reflecting greater symptoms of depression. A cut-off score of 10 represents significant depressive symptoms [31]. The CESD-10 has been well-validated and has demonstrated good internal consistency when assessed within a one-week timeframe [31]. The Cronbach’s alpha for the current study was 0.81.

*Health Behaviors*. Consumption of high-fat foods and fruits and vegetables was assessed using two items from the Cardiovascular Risk Assessment (CRA; [32]). Women were asked to report the average number of times they consumed high-fat foods (e.g., snacks, chips, and fried foods) per week during their pregnancy on a 4-point scale of 1 (‘Never or rarely’) to 4 (‘6 or more times/week’), as well as the average number of servings of fruits and vegetables they consumed per week on a 4-point scale of 1 (‘None or less than 1 serving/week’) to 4 (‘6 or more servings/week’). A third CRA item measured the type (e.g., brisk walking) and duration of moderate-intensity exercise that women engaged in during pregnancy, with higher scores representing greater minutes of moderate-intensity exercise per week. Finally, a fourth item measured the type (e.g., diaphragmatic breathing) and duration of stress management that women engaged in during pregnancy, with higher scores representing greater minutes of stress management engaged in per week.

*Qualitative Interview*. During the health interview, participants were asked the following open-ended questions to gather contextual information on the types of stressors that women experienced and the health behaviors they engaged in during pregnancy:

1. How has being pregnant affected your eating habits?
2. What type of exercise have you performed, if any, during your pregnancy?
3. What forms of stress management have you used, if any, during your pregnancy?

### 2.4. Statistical Analyses

An a priori power analysis, using G*Power [33], estimated that approximately 88 participants would be needed to achieve statistical power at the recommended 0.80 level with an alpha level of 0.05. Possible covariates affecting study outcomes were identified using Pearson correlations and ANOVAs. Only age, income, and ethnicity were significantly associated with women’s prenatal health behaviors (*p* < 0.05) and were included as covariates in subsequent analyses. Four separate hierarchical regression analyses were performed to identify which demographic (i.e., age, income, or ethnicity) and psychosocial characteristics (i.e., coping, stress, pregnancy-specific stress, or depression) were associated with each health behavior (i.e., high-fat food consumption, fruit and vegetable consumption, exercise, and stress management). The effect sizes for these associations were presented as Cohen’s *f*^2^ with values of 0.02, 0.15, and 0.35 representing a small, medium, and large effect size, respectively [34]. Ethnicity was dummy-coded (ethnic minority vs. non-Hispanic white) in the regression models with post hoc ANCOVA analyses conducted to more closely examine group differences in prenatal health behaviors by ethnicity (e.g., foreign-born Latina vs. U.S.-born Latina), controlling for age and income.

Qualitative analyses of interview transcripts were conducted using an inductive thematic analysis approach [35]. Potential themes and subthemes were developed from marginal codes, which were used as a codebook. Two independent coders pilot-tested the codebook on 50% of the focus group transcripts. Further revisions to codes and definitions were made to accurately capture participants’ experiences. During the second phase of analyses, two independent coders were paired and utilized the codebook created in the first phase to apply codes to excerpts. Any disagreements on the application of codes were resolved through a group discussion until a consensus was reached. The last half of the coded transcripts was assessed for the level of agreement in codes applied using Cohen’s kappa. Interrater reliability was strong (*k* = 0.80).

## 3. Results

### 3.1. Participants

Participants were approximately 26 years of age (range = 18–45 years), in their 17th week of pregnancy (range = 10–24 weeks), and had one child on average (range = 0–7 children). Most women were single (51%), with a high-school education or less (61%) and a total family annual income under USD 25,000 (75%). In terms of ethnicity, 39% were African American (all U.S.-born), 30% were foreign-born Latinas (77% from Mexico), 15% were U.S.-born Latinas, 10% were non-Hispanic white, and 6% were Asian American/Pacific Islander (33% from Cambodia, 33% from the Philippines). Foreign-born Latinas tended to be older [*F*(4, 96) = 4.98, *p* = 0.001], have more children [*F*(4, 96) = 4.01, *p* = 0.01], and have a higher body mass index (BMI) [*F*(4, 96) = 3.18, *p* = 0.02] compared to most other ethnic groups, while African American women were less likely to be married or living together with their partner [*χ*^2^(4, 96) = 26.87, *p* < 0.001]. Further, non-Hispanic white and Asian American/Pacific Islander women were more likely to have a higher income [*χ*^2^(4, 96) = 31.95, *p* < 0.001] and education level [*χ*^2^(4, 96) = 18.84, *p* < 0.001] than other ethnic groups (see Table 1 for demographic characteristics by ethnicity). Approximately 24% of all women experienced elevated stress (PSS-4 score > 8) and 42% had high depressive symptoms (CESD-10 score > 10). Notably, foreign-born Latinas experienced more pregnancy-related stress [*F*(4, 96) = 3.03, *p* = 0.03] and engaged in less planning to cope [*F*(4, 96) = 8.84, *p* < 0.001] compared to all other ethnic groups (see Table 1 for psychosocial characteristics by ethnicity).

### 3.2. Factors Associated with Prenatal Health Behaviors

**Figure 1:**
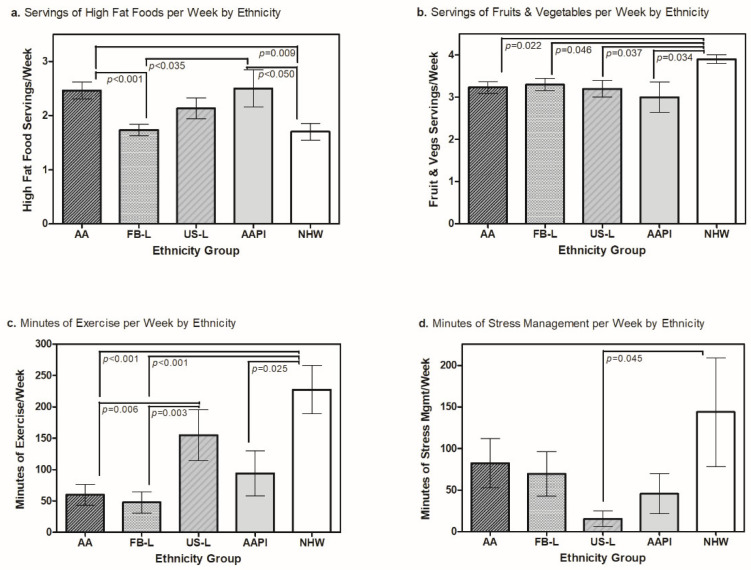
Women’s prenatal health behaviors (nutrition, exercise, and stress management) by ethnicity. AA = African American; FB-L = foreign-born Latina; US-L = U.S.-born Latina; AAPI = Asian American and Pacific Islander; NHW = non-Hispanic white.

*Nutrition*. Approximately one-third of women (31%) reported consuming three or more servings of high-fat foods per week, and 51% consumed less than five servings of fruits and vegetables per week. Younger women (ΔR^2^ = 0.10; *p* = 0.001), those who used more avoidance coping (ΔR^2^ = 0.05; *p* = 0.001), and those with elevated stress levels (ΔR^2^ = 0.05; *p* = 0.027) were found to consume more high-fat foods (see Table 2). Ethnicity was also associated with high-fat food consumption (ΔR^2^ = 0.04; *p* = 0.029), with African American and Asian American/Pacific Islander women consuming more high-fat foods than foreign-born Latinas and non-Hispanic white women [*F*(4, 96) = 4.55, *p* = 0.002; see Figure 1a]. The overall variance explained using the final model was 29% (*f*^2^ = 0.42). Of note, no factors were significantly associated with fruit and vegetable consumption (*R*^2^ = 0.11, *p* = 0.392, *f*^2^ = 0.12), although post hoc ANOVA analyses demonstrated that non-Hispanic white women consumed more fruits and vegetables than all other ethnic groups [*F*(4, 96) = 1.70, *p* = 0.022; see Figure 1b].

When asked about their eating habits during pregnancy, 95% of the women (74% ethnic minority) described changes in their food cravings while pregnant, with some women indicating that they had new cravings for healthy snacks while others craved high-fat foods (see Table 3). “*I don’t crave it [fast food/high-fat foods]. If I do crave it, I will go ahead and do it, but if I know that I have had too much of it, I just won’t. I would rather munch on something like grapes.*” Another woman shared, “*Yeah it [being pregnant] made me want to eat more junk [food]. Stuff I wouldn’t normally eat, I eat now. Like a peanut butter and jelly sandwich, I hate peanut butter and jelly.*” Further, 30% of women described being motivated to eat healthier for themselves and their babies. “*After the pregnancy, I am planning to breastfeed, and I heard that if you eat certain foods then it gets passed on to the baby.*”

*Exercise*. Approximately 40% of women reported not exercising during pregnancy, with only 23% meeting the national recommendation of engaging in 150 min or more of moderate-intensity exercise per week (*M* = 94.23, *SD* = 130.81). Of women that exercised, the two most common forms of physical activity were brisk walking (52%) and heavy housework (20%; see Table 4). Women with a lower annual household income (ΔR^2^ = 0.07; *p* = 0.014), those who used less planning to cope (ΔR^2^ = 0.11; *p* = 0.001), and those who experienced more intense pregnancy-specific stressors (ΔR^2^ = 0.02; *p* = 0.048) exercised less during pregnancy (see Table 2). Ethnicity was also associated with exercise (ΔR^2^ = 0.02; *p* = 0.049), with African American women, foreign-born Latinas, and Asian American/Pacific Islanders exercising less than U.S.-born Latinas and non-Hispanic white women [*F*(4, 96) = 6.34, *p* < 0.001; see Figure 1c]. The overall variance explained using the final model was 25% (*f*^2^ = 0.34).

When asked about their exercise behavior during pregnancy, 63% of women (76% ethnic minority) described exercising less during pregnancy due to experiencing morning sickness, feeling too tired or fatigued, and having fears about what exercising would do to the health of the baby (see Table 3). “*The pregnancy has affected my exercise a lot because I feel drained sometimes and I sleep a lot. I just feel like I don’t want to get up.*” Another mother discussed her fear of exercising: “*And all the lifting. No, I can’t do that because I’m really afraid that it’s going to do something to myself and to the baby.*” Approximately 90% of women (75% ethnic minority) reported being informed about prenatal exercise, with one mother describing that she began exercising during pregnancy because of her doctor’s recommendation, “*The doctor told me that I needed to exercise. And then I also heard that exercise [increases] stamina. I decided to exercise more.*” Finally, 48% mentioned that there were health benefits to exercising while pregnant. “*I also heard about walking. If you walk a lot, it helps you with the labor.*”

*Stress Management*. Only 40% of women reported participating in some form of relaxation during pregnancy (*M* = 72.18 min/week, *SD* = 155.12), with 21% engaging in empirically supported forms of stress management [massage (9%), breathing techniques (8%), and meditation (8%)] and 19% engaging in other self-care activities [praying (5%), taking a bath/shower (5%), and taking a nap (3%); see Table 4]. Younger women (ΔR^2^ = 0.08; *p* = 0.006) and those who used less avoidance coping (ΔR^2^ = 0.05; *p* = 0.016) engaged in less stress management during pregnancy (see Table 2). Ethnicity was also associated with stress management (ΔR^2^ = 0.02; *p* = 0.049), with U.S.-born Latinas using fewer forms of relaxation than non-Hispanic white women [*F*(4, 96) = 1.75, *p* = 0.045; see Figure 1d]. The overall variance explained using the final model was 19% (*f*^2^ = 0.23).

When asked about their engagement in stress management during pregnancy, 50% of women (75% ethnic minority) reported learning about different forms of relaxation through their social networks, personal experiences, or their health care providers (see Table 3). “*So there’s Lamaze, there’s hypno-breathing, there’s all these different types of ways to do natural birth, and this is the technique [relaxation breathing] that my midwife recommends.*” Of those that used stress management, 50% described the health benefits they experienced, including feeling more relaxed, noting improvements in their emotional and spiritual well-being, and having less muscle tension. “*After I practice a breathing technique or a relaxation technique, I definitely feel a lot better afterwards as far as relaxed and calm. Almost the same as taking a mini break.*” Furthermore, 75% of women (77% ethnic minority) described wanting to learn more about the benefits of stress management during pregnancy: “*I want to learn more about muscle relaxation because I am stressed from my neck and from my muscles in my shoulders all the time.*”

## 4. Discussion

The current mixed-methods study is one of the few to identify factors associated with the prenatal health behaviors of low-income, ethnic minority women in southern California. Overall, our findings indicate that a substantial proportion of women (31% to 77%) are engaging in unhealthy prenatal health behaviors. Specifically, younger ethnic minority women, with high levels of stress and the use of maladaptive coping strategies, are particularly at risk for not meeting the ACOG’s prenatal health behavior guidelines [5,7].

### 4.1. Nutrition

Regarding nutrition, results indicated that about one-third of pregnant women consumed high-fat foods three or more times per week, with African American and Asian American/Pacific Islander women consuming more high-fat foods than foreign-born Latinas and non-Hispanic white women. Although these results are consistent with previous studies of African American pregnant women [18,24], this is one of the first studies to show that Asian American/Pacific Islanders are also at risk for high-fat food consumption during pregnancy and highlights the need to examine factors such as food insecurity, access to healthy foods, U.S. acculturation, and cultural and taste preferences for certain foods affecting prenatal nutrition habits in these communities [36]. Stress and avoidance coping also emerged as key factors associated with high-fat food consumption. Previous studies have shown that experiencing chronic stressors may result in increased production of the stress hormone cortisol, which has been linked with increasing one’s cravings for high-fat foods and sweets [37]. Additionally, stressors perceived as threatening and uncontrollable have been associated with the use of more avoidant coping techniques as a means of escaping from having to deal with these stressors, resulting in higher rates of alcohol use, sedentary behavior, and postpartum depression [38,39]. This is the first study to our knowledge to suggest that pregnant women’s use of avoidance coping is linked to higher consumption of high-fat foods, emphasizing the need to screen for the presence of chronic stressors and avoidant coping behaviors during pregnancy.

### 4.2. Exercise

Less than 25% of women met national recommendations for exercise during pregnancy, with African American women, foreign-born Latinas, and Asian American/Pacific Islanders exercising less than U.S.-born Latinas and non-Hispanic white women. Our qualitative findings suggest that these lower exercise rates are due to experiencing morning sickness, feeling fatigued, and being fearful that exercise may adversely affect the health of the baby. These results are similar to those showing low-income African American women to be fearful of exercising while pregnant because it may harm their babies [40]. Certain Asian subgroups, such as those from China, have also reported cultural norms of not overexerting oneself during pregnancy to prevent birth complications [41]. For foreign-born Latinas, previous studies have shown that acculturation may impact exercise behaviors during pregnancy and the early postpartum period. Specifically, studies have indicated that they may be more likely to care for a greater number of dependent children and be influenced by the cultural value of familismo (emphasis on the importance of the family) more than other ethnic groups and, therefore, have competing childcare obligations and stress related to their maternal roles that serve as unique barriers to exercise in this population [42,43]. Together, these results underscore the importance of having prenatal health care providers discuss the benefits of exercise with ethnic minority women and their families to promote the health of their babies. Results also revealed that foreign-born Latinas experienced more pregnancy-related stress (e.g., physical discomfort and fatigue) and used less planning to cope with their stress than other ethnic groups—factors associated with exercising less during pregnancy in this and other studies [25,44]. Therefore, physical activity interventions are indicated that can facilitate stress management to improve prenatal exercise behavior in this at-risk population.

### 4.3. Stress Management

The ACOG has prominently endorsed the use of cognitive behavioral stress management (CBSM) approaches (e.g., diaphragmatic breathing and muscle relaxation) as one of the best non-pharmacological methods and empirically-supported modalities for helping to prevent stress-related disorders during pregnancy. Despite these recommendations, results revealed that less than one-fourth of women in our sample used CBSM, with 19% engaging in other self-care activities (e.g., prayer or taking a bath/nap) to manage their stress. U.S.-born Latinas used less stress management than non-Hispanic white women, consistent with previous studies demonstrating Latinas (particularly Mexican Americans) to be less confident in managing their stress and more likely to use natural herbs and vitamins for stress management due to cultural differences in health and illness beliefs, with these alternate remedies being more natural and accepted ways to treat common physical symptoms of stress (e.g., nerves, headaches, insomnia, and chest pain; [45,46]). Additionally, younger women and those using less avoidance coping engaged in less stress management, possibly reflecting their lack of experience or awareness of the efficacy of CBSM strategies in managing their stress or of ways to implement them. Although most women in our sample (79%) had never engaged in CBSM, qualitative results revealed that 75% of women were interested in learning more about these stress management approaches, thereby providing support for translational interventions that target the dissemination of CBSM information among prenatal health care providers.

### 4.4. Study Limitations

Several limitations merit mention. First, although our sample was ethnically diverse, the results may not be generalizable or represent the experiences of women from other ethnic backgrounds or other parts of the world. Therefore, additional studies in this research area, with larger sample sizes, are indicated to support our findings and further our understanding of prenatal health behaviors across several ethnic subgroups. Second, the coping measures used were specific to pregnancy-related stressors. Future studies may want to assess coping resources used to manage chronic stressors experienced by different ethnic groups (e.g., coping with discrimination and/or acculturation) to determine their association with observed differences in prenatal health behaviors. Third, this study did not assess for acculturation level, which may be more effective in examining cultural factors (e.g., health beliefs) that may impact women’s prenatal health behaviors when compared to ethnicity or birthplace alone. Finally, this study was cross-sectional by design, and therefore any predictive interpretations were not feasible.

## 5. Conclusions

In sum, our findings indicate that a substantial proportion of women are engaging in unhealthy prenatal health behaviors. Preventive and treatment interventions are needed that address both systemic (e.g., safe exercise locations for low-income families) and individual-level factors (e.g., healthy coping patterns) to help women manage the demands and challenges associated with pregnancy and motherhood. These interventions should be culturally tailored to address cultural beliefs and customs that might influence health behaviors. Additionally, a collaborative care model is needed to support prenatal health providers in linking their patients to available stress management and social work services [47]. Finally, additional training is needed to support health care and community workers in the delivery of stress management interventions that can teach women effective coping strategies for incorporating prenatal health behaviors [48].

## Figures and Tables

**Table 1:** Demographic and psychosocial characteristics among pregnant women by ethnicity.

	Total Sample *n* = 100	African American *n* = 39	Foreign-Born Latinas *n* = 30	U.S.-Born Latinas *n* = 15	Asian American/Pacific Islander *n* = 6	Non-Hispanic White *n* = 10
**Demographic**						
Age [M (SD)] **	25.8 (± 6.0)	23.8 (± 5.6)	28.7 (± 5.7) *	22.2 (± 3.4)	27.3 (± 5.1)	28.0 (± 7.6) *
Gestational Age *[M (SD)]*	17.0 (± 4.8)	16.9 (± 5.0)	16.9 (± 4.6)	18.2 (± 4.2)	16.5 (± 6.0)	17.7 (± 5.4)
BMI [M (SD)] *	27.7 (± 5.9)	27.5 (± 5.9)	29.4 (± 6.0) **	29.8 (± 6.5) **	24.4 (± 3.2)	23.2 (± 2.8)
Number of Children [M (SD)] **	1.1 (± 1.5)	1.0 (± 1.5)	1.9 (± 1.7) **	0.5 (± 0.8)	0.5 (± 0.8)	0.4 (± 0.7)
Married/Living Together (%) ***	49.0	14.3 ***	76.7	53.3	66.7	70.0
High-school education or less (%) **	61.0	64.1	83.3	53.3	16.7 **	20.0 **
Annual family combined income < USD 25,000 (%) ***	75.0	87.2	93.3	60.0	33.3 ***	20.0 ***
**Psychosocial** **Characteristics**						
Planning [M (SD)] ***	22.8 (± 6.6)	25.8 (± 5.0)	18.5 (± 6.7) **	23.6 (± 7.2)	21.7 (± 5.6)	23.0 (± 4.8)
Avoidance *[M (SD)]*	14.5 (± 4.3)	14.6 (± 4.8)	14.4 (± 3.8)	14.3 (± 4.9)	15.3 (± 4.7)	14.1 (± 4.0)
Social Support *[M (SD)]*	32.5 (± 6.9)	31.6 (± 7.1)	33.0 (± 5.9)	33.2 (± 7.7)	32.8 (± 7.2)	33.1 (± 8.0)
Stress *[M (SD)]*	5.2 (± 3.2)	5.2 (± 3.5)	5.2 (± 2.4)	5.4 (± 3.8)	5.7 (± 4.0)	5.0 (± 2.9)
Frequency Pregnancy Stress [M (SD)] *	0.8 (± 0.6)	0.7 (± 0.4)	1.0 (± 0.8) *	0.7 (± 0.4)	0.7 (± 0.3)	0.7 (± 0.3)
Intensity Pregnancy Stress *[M (SD)]*	0.7 (± 0.3)	0.7 (± 0.3)	0.7 (± 0.3)	0.6 (± 0.3)	0.7 (± 0.2)	0.8 (± 0.2)
Depression *[M (SD)]*	9.2 (± 6.2)	10.6 (± 7.1)	9.3 (± 5.5)	6.6 (± 5.4)	7.8 (± 3.9)	7.7 (± 5.1)

Note. MANOVAs and Pearson’s *χ*^2^ were conducted to test for group differences (ethnicity) in demographic and psychosocial characteristics. *M* = mean; *SD* = standard deviation. * *p* < 0.05; ** *p* < 0.01; *** *p* < 0.001.

**Table 2:** Hierarchical regression analyses for factors associated with prenatal health behaviors (*N* = 100).

	High-Fat Foods			Fruits and Vegetables			Exercise			Stress Management		
Variables	*R* ^2^	Standard *β*	Standard Error	*R* ^2^	Standard *β*	Standard Error	*R* ^2^	Standard *β*	Standard Error	*R* ^2^	Standard *β*	Standard Error
	0.29 ***			0.11			0.25 **			0.19 *		
Age		−0.32 **	0.01		−0.03	0.02		−0.06	2.24		0.28 **	2.5
Income		0.07	0.04		0.18	0.05		0.26 *	5.9		0.16	8.45
Ethnicity		−0.25 *	0.09		0.01	0.09		0.23 *	12.53		−0.22 *	17.31
Planning		0.08	0.02		−0.04	0.02		0.36 **	1.91		−0.21	2.92
Avoidance		0.39 **	0.02		0.04	0.02		0.06	3.3		0.30 *	4.36
Social Support		0.01	0.01		0.15	0.02		0.11	2.14		−0.15	2.78
Stress		0.32 *	0.04		−0.09	0.04		−0.03	5.6		0.09	7.45
Frequency Pregnancy Stress		−0.02	0.16		−0.02	0.17		−0.05	23.86		0.03	31.33
Intensity Pregnancy Stress		0.05	0.38		0.07	0.41		−0.24 *	57.9		0.05	74.79
Depression		−0.07	0.02		−0.05	0.02		0.24	3		−0.23	3.77

* *p* < 0.05; ** *p* < 0.01; *** *p* < 0.001.

**Table 3:** Qualitative themes for women’s prenatal health behaviors (*n* = 40).

Main Theme	*N* *	Definition	Sample Quote
**Subtheme**			
**How have your eating habits changed?**			
New food cravings	121	Participants mentioned new cravings during pregnancy that they did not have previously.	“*I never bought ice cream until now. I probably haven’t bought ice cream in five years.*”
No change	21	Participants mentioned having no changes in eating habits or cravings.	“*Well, it [pregnancy] has not affected me because it is the same. I continue eating the same.*”
**How has pregnancy affected your exercise?**			
Decreased exercise	33	Participants mentioned a decrease in the amount of exercise they engaged in during pregnancy.	“*I used to be able to do a lot of stuff, and after giving birth, I kind of really can’t do it anymore.*”
Increased exercise	8	Participants mentioned an increase in the amount of exercise they engaged in during pregnancy.	“*I’ve stepped up swimming. I didn’t do it as often, but it’s something that’s very nice to do now.*”
Advised to exercise	76	Participants mentioned the ways they were informed about exercising during pregnancy.	“*My doctor said that prenatal yoga is a really good way to stretch and keep your muscles loose.*”
Learned benefits to exercise	30	Participants mentioned the benefits they learned from engaging in exercise during pregnancy.	“*Well, I used to have a lot of back problems and now I don’t notice it as much.*
**What forms of stress management used?**			
Sources of stress management	46	Participants mentioned where they learned about or were taught about some of the relaxation exercises in which they engaged.	“*I heard about breathing because I took my nursing classes.*”
Learned benefits to stress management	37	Participants mentioned some of the benefits they experienced after engaging in relaxation exercises during pregnancy.	“*I’ll buy a relaxing CD and then play it when I’m in the bath or just home alone.*”
Interest in learning more about stress management	45	Participants mentioned some of the relaxation exercises they were interested in learning more about.	“*Muscle relaxation. I’ve never heard of that. I mean I’ve heard of it, but I haven’t tried it. I guess that would be something new.*”

* Note. Numbers represent the number of instances that a code was discussed by participants.

**Table 4:** Prevalence of women’s exercise and stress management behaviors during pregnancy (*N* = 100).

	% Endorsing *
**Types of Exercise**	
Brisk walking	52
Heavy housework	20
Aerobic fitnesss	10
Leisure-recreational sports	5
Yardwork	5
Bicycling	4
Jogging/running	3
Competitive sports	3
Strength training	1
Swimming	1
Badminton	1
**Types of Stress Management**	
Massage	9
Diaphragmatic breathing	8
Meditation	8
Muscle relaxation	4
Aromatherapy	3
Visualization	2
Acupuncture	1
**Other Self-Care/Relaxation Activities**	
Praying	5
Bathing/showering	5
Sleeping/napping	3
Listening to music	3
Reading	2
Sewing	1
Watching TV	1
Eating ice chips	1
Positive thoughts about baby	1
Going on a walk	1

* Note. Percentages include those who endorsed more than one form of exercise or stress management.

## Data Availability

The data that support the findings of this study are available from the corresponding author [GU] upon reasonable request.

## References

[1] Chia A.R., Chen L.W., Lai J.S., Wong C.H., Neelakantan N., van Dam R.M., Chong M.F. (2019). Maternal dietary patterns and birth outcomes: A systematic review and meta-analysis. Adv. Nutr..

[2] Jones M.A., Catov J.M., Jeyabalan A., Whitaker K.M., Barone Gibbs B. (2021). Sedentary behaviour and physical activity across pregnancy and birth outcomes. Paediatr. Perinat. Epidemiol..

[3] Kibret K.T., Chojenta C., Gresham E., Tegegne T.K., Loxton D. (2019). Maternal dietary patterns and risk of adverse pregnancy (hypertensive disorders of pregnancy and gestational diabetes mellitus) and birth (preterm birth and low birth weight) outcomes: A systematic review and meta-analysis. Public Health Nutr..

[4] Littleton H.L., Bye K., Buck K., Amacker A. (2010). Psychosocial stress during pregnancy and perinatal outcomes: A meta-analytic review. J. Psychosom. Obstet. Gynaecol..

[5] American College of Obstetricians & Gynecologists (2020). Physical activity and exercise during pregnancy and the postpartum period: ACOG Committee Opinion, Number 804. Obstet. Gynecol..

[6] Kominiarek M.A., Rajan P. (2016). Nutrition recommendations in pregnancy and lactation. Med. Clin. N. Am..

[7] American College of Obstetricians and Gynecologists Committee on Health Care for Undeserved Women (2006). ACOG Committee Opinion No. 343: Psychosocial risk factors: Perinatal screening and intervention. Obstet. Gynecol..

[8] Abu-Saad K., Fraser D. (2010). Maternal nutrition and birth outcomes. Epidemiol. Rev..

[9] Fink N.S., Urech C., Cavelti M., Alder J. (2012). Relaxation during pregnancy: What are the benefits for mother, fetus, and the newborn? A systematic review of the literature. J. Perinat. Neonatal Nurs..

[10] Moyer C., Reoyo O.R., May L. (2016). The Influence of prenatal exercise on offspring health: A review. Clin. Med. Insights. Women’s Health.

[11] Francis E.C., Zhang L., Witrick B., Chen L. (2021). Health behaviors of American pregnant women: A cross-sectional analysis of NHANES 2007–2014. J. Public Health.

[12] Hesketh K.R., Evenson K.R. (2016). Prevalence of U.S. pregnant women meeting 2015 ACOG physical activity guidelines. Am. J. Prev. Med..

[13] Birdee G.S., Kemper K.J., Rothman R., Gardiner P. (2014). Use of complementary and alternative medicine during pregnancy and the postpartum period: An analysis of the National Health Interview Survey. J. Women’s Health.

[14] Cox E.Q., Sowa N.A., Meltzer-Brody S.E., Gaynes B.N. (2016). The perinatal depression treatment cascade: Baby steps toward improving outcomes. J. Clin. Psychiatry.

[15] Blumenshine P., Egerter S., Barclay C.J., Cubbin C., Braveman P.A. (2010). Socioeconomic disparities in adverse birth outcomes: A systematic review. Am. J. Prev. Med..

[16] Evenson K.R., Wen F. (2010). National trends in self-reported physical activity and sedentary behaviors among pregnant women: NHANES 1999–2006. Prev. Med..

[17] Fox M., Entringer S., Buss C., DeHaene J., Wadhwa P.D. (2015). Intergenerational transmission of the effects of acculturation on health in Hispanic Americans: A fetal programming perspective. Am. J. Public Health.

[18] Hill A.M., Nunnery D.L., Ammerman A., Dharod J.M. (2020). Racial/ethnic differences in diet quality and eating habits among WIC pregnant women: Implications for policy and practice. Am. J. Health Promot..

[19] Larrabee Sonderlund A., Schoenthaler A., Thilsing T. (2021). The Association between maternal experiences of interpersonal discrimination and adverse birth outcomes: A systematic review of the evidence. Int. J. Environ. Res. Public Health.

[20] Park C.L., Iacocca M.O. (2014). A stress and coping perspective on health behaviors: Theoretical and methodological considerations. Anxiety Stress Coping.

[21] Alhusen J.L., Ayres L., DePriest K. (2016). Effects of maternal mental health on engagement in favorable health practices during pregnancy. J. Midwifery Women’s Health.

[22] Auerbach M.V., Lobel M., Cannella D.T. (2014). Psychosocial correlates of health-promoting and health-impairing behaviors in pregnancy. J. Psychosom. Obstet. Gynaecol..

[23] Cannella B.L., Yarcheski A., Mahon N.E. (2018). Meta-analyses of predictors of health practices in pregnant women. West. J. Nurs. Res..

[24] Harris A., Chilukuri N., West M., Henderson J., Lawson S., Polk S., Levine D., Bennett W.L. (2016). Obesity-related dietary behaviors among racially and ethnically diverse pregnant and postpartum women. J. Pregnancy.

[25] Pasha H., Faramarzi M., Chehrazi M., Bakouei F., Gholinia H., Abdollahi S., Shafierizi S. (2021). Health-promotion and health-harming behaviours in pregnant women: Role of coping strategies, anxiety, and depression. J. Obstet. Gynaecol..

[26] U.S. Census Bureau (2014). Selected Economic Characteristics. 2010–2014 American Community Survey 5-Year Estimates. https://www.census.gov/programs-surveys/acs/technical-documentation/table-and-geography-changes/2014/5-year.html.

[27] Yali A.M., Lobel M. (2002). Stress-resistance resources and coping in pregnancy. Anxiety Stress Coping.

[28] Baker D., Taylor H. (1997). The relationship between condition-specific morbidity, social support and material deprivation in pregnancy and early motherhood. ALSPAC Survey Team. Avon Longitudinal Study of Pregnancy and Childhood. Soc. Sci. Med..

[29] Cohen S., Williamson G., Spacapam S., Oskamp S. (1988). Perceived stress in a probability sample of the United States. The Social Psychology of Health: Claremont Symposium on Applied Social Psychology.

[30] DiPietro J.A., Christensen A.L., Costigan K.A. (2008). The pregnancy experience scale-brief version. J. Psychosom. Obstet. Gynaecol..

[31] Cole J.C., Rabin A.S., Smith T.L., Kaufman A.S. (2004). Development and validation of a Rasch-derived CES-D short form. Psychol. Assess..

[32] Danhauer S.C., Oliveira B., Myll J., Berra K., Haskell W. (2004). Successful dietary changes in a cardiovascular risk reduction intervention are differentially predicted by biopsychosocial characteristics. Prev. Med..

[33] Faul F., Erdfelder E., Buchner A., Lang A.G. (2009). Statistical power analyses using G*Power 3.1: Tests for correlation and regression analyses. Behav. Res. Methods.

[34] Cohen J. (1988). Statistical Power Analysis for the Behavioral Sciences.

[35] Braun V., Clarke V. (2006). Using thematic analysis in psychology. Qual. Res. Psychol..

[36] Henry C.J., Kaur B., Quek R. (2020). Are Asian foods as “fattening” as western-styled fast foods?. Eur. J. Clin. Nutr..

[37] Epel E., Lapidus R., McEwen B., Brownell K. (2001). Stress may add bite to appetite in women: A laboratory study of stress-induced cortisol and eating behavior. Psychoneuroendocrinology.

[38] Doron J., Trouillet R., Maneveau A., Ninot G., Neveu D. (2015). Coping profiles, perceived stress and health-related behaviors: A cluster analysis approach. Health Promot. Int..

[39] Guardino C.M., Schetter C.D. (2014). Coping during pregnancy: A systematic review and recommendations. Health Psychol. Rev..

[40] Groth S.W., Morrison-Beedy D. (2013). Low-income, pregnant, African American women’s views on physical activity and diet. J. Midwifery Women’s Health.

[41] Lee D.T., Ngai I.S., Ng M.M., Lok I.H., Yip A.S., Chung T.K. (2009). Antenatal taboos among Chinese women in Hong Kong. Midwifery.

[42] Urizar G.G., Hurtz S.Q., Ahn D.K., King A.C., Albright C.L., Atienza A.A. (2005). Influence of maternal stress on successful participation in a physical activity intervention: The IMPACT Project. Women Health.

[43] Vermeesch A.L., Stommel M. (2014). Physical activity and acculturation among U.S. Latinas of childbearing age. West. J. Nurs. Res..

[44] Lobel M., Cannella D.L., Graham J.E., DeVincent C., Schneider J., Meyer B.A. (2008). Pregnancy-specific stress, prenatal health behaviors, and birth outcomes. Health Psychol..

[45] Suarez-Cano G. (2018). Racial and Ethnic Differences in Perceived Stress, Social Support, and Stress Management. Master’s Thesis.

[46] Upchurch D.M., Wexler Rainisch B.K. (2012). Racial and ethnic profiles of complementary and alternative medicine use among young adults in the United States: Findings from the National Longitudinal Study of Adolescent Health. J. Evid.-Based Complement. Altern. Med..

[47] Siu A.L., Bibbins-Domingo K., Grossman D.C., Baumann L.C., Davidson K.W., Ebell M., García F.A., Gillman M., Herzstein J., US Preventive Services Task Force (USPSTF) (2016). Screening for depression in adults: US preventive services task force recommendation statement. JAMA.

[48] Urizar G.G., Yim I.S., Rodriguez A., Schetter C.D. (2019). The SMART Moms Program: A Randomized Trial of the Impact of Stress Management on Perceived Stress and Cortisol in Low-Income Pregnant Women. Psychoneuroendocrinology.

